# Clinical Trial Assessment Principles of National Class III Medical Devices in China

**DOI:** 10.1111/os.12498

**Published:** 2019-09-06

**Authors:** Yue Min, Jin‐tao Gao, Jing Wu, Bao Zhai, Dan Han, Bin Liu

**Affiliations:** ^1^ Center for Medical Device Evaluation, Center for Medical Device Evaluation NMPA Beijing China

**Keywords:** Assessment indices, Class III medical device, Clinical trial, Data analysis

## Abstract

Class III medical devices are defined as those which are implanted inside the human body and applied to maintain normal life and retain original tissue or organic functions. Because these devices are associated with high risk, their effectiveness and safety should be strictly monitored and clinically investigated. The aim of clinical investigation of these medical devices is to ensure the acceptability of their effectiveness and safety levels. On designing the clinical trial, the investigator should determine the indices to assess the effectiveness and safety of medical devices, select reasonable data‐analyzing methods, and pay attention to several other issues. Although some guidelines on specific class III medical devices have illustrated those aspects in detail, there is still no comprehensive report that details all those principles and methodologies. This article aims to summarize the common features among the instruction principles and provide technological support for the clinical study of class III medical devices.

## Introduction

As class III medical devices present the highest risk among medical devices for patients in China, the management and control of those devices is much more stringent than for other devices^1^. However, it should be noted that there is an obvious difference between the guidelines followed in China and in other countries, such as the USA. In the USA, clinical trials are not required for FDA approval of medical devices^2^. Furthermore, sometimes even preclinical animal model tests are not performed for grafted products such as demineralized bone matrix (DBM), because the FDA does not regard this as a necessary procedure^3^. In China, if existing clinical evidence is not sufficient to assure the effectiveness and safety of a medical device, a clinical investigation might be necessary. In addition, the clinical trial should be planned and conducted properly, with appropriate analysis and reporting^4^.

When clinically testing and examining class III medical devices, the investigator should first verify all information on the device relating to its clinical application, including the proper range of application, contraindications, methods of application, warnings, and announcements. On assessing the clinical trials, the investigator should focus on relative guiding principles, and fully recognize the possible situations for relevant trials. The assessment methodology of clinical trials usually involves comparison of the device with another similar device that has already been applied in the market, to evaluate the effectiveness and safety in its prospective applications. For most class III medical devices to be registered (such as CaP/CaSi bone‐filling materials), a non‐inferiority clinical trial needs to be conducted. The trial indicates the effectiveness of the medical device within a certain range comparable to a control group product^5^.

Besides what is mentioned above, the investigator should also determine the ideal indices to assess medical devices, select reasonable data‐analyzing methods, and pay attention to several other announcements on conducting the clinical trials. Some guidelines on specific class III medical devices have illustrated those aspects in detail. Nonetheless, to the best of the author's knowledge, there is no comprehensive report that has included those principles together. This article aims to summarize the common features among the instruction principles, to compare the differences, and to provide technological support for the clinical trials and assessments of class III medical devices.

## Selection of Clinical Trial Assessment Indices

Selecting appropriate assessment indices is essential for determining the sample size and for effective evaluation of the results in clinical trials^6^. The indices of class III medical devices can usually be divided into effectiveness indices and safety indices; the former can be further divided into major and minor indices. The investigator should set the most representative index, which directly reflects the prospective functions and effects of the device, as the major index. The assessment is usually based on results of quantitative analyses of the parameters. The minor indices could include any other available aspects, such as the postoperative morphology, stability and the Oswestry disability index (ODI) (as variates of time). Noticeably, although those minor indices are considered minor, they are still indispensable as a part of the final trial results. Safety indices emphasize the safety of the product materials inside the human body, and mainly reveal any types of adverse reactions, including severe adverse reactions. Table 1 lists several examples of assessment indices for specific class III medical devices, stated in relative medical device guidelines. The investigator should determine whether an index of effectiveness or safety can be considered more essential than another, based on the properties and prospective application of the product. The investigator should also illustrate the reason for selecting the major index. In designing the clinical trial, the investigator should ascertain the assessment methods, the applied factors of single or multiple variates, and the relative parameters of each index. Finally, the investigator needs to verify whether the product performs well in patients using quantitative or qualitative methods as stated in the relevant guidelines.

**Table 1:** Examples of class III medical devices and their assessment indices

Class III medical devices	Efficacy indices		Safety indices	References
	Major	Minor		
CaP/CaSi bone‐filling materials	Fusion rate at the imagological endpoint	Bone defect cure time, bone‐filling material resorption rate, new bone formation rate, bone density, ODI etc.	Cure of cut in follow‐up period, rejecting reaction, subjective feelings of patients etc.	1
Artificial cervical intervertebral disc prosthesis	Success rate of treatment at the 12th month after operation	JOA, radiographic assessment	Prosthetic survival rate, adverse reaction occurrence rate, and complication rate	3
Posterior spinal products for internal fixation	The lateral position, flexion, and extension of the X‐ray and reconstruction CT to evaluate the situation of deformation, deviation, loosening, and fracture	Bone fusion or cure of bone fracture at fixed segments, JOA, ODI etc.	n/a	4
Artificial cochlea	Free field hearing threshold, improvement of speech recognition in quiet environment	Assessment from the doctor on the properties of artificial cochlea	Blood routine examination, hepatorenal function, inflammatory response, and abnormal working of the device	2

JOA, Japanese Orthopaedic Association score; ODI, Oswestry disability index.n/a, not available.

## Statistical Analysis of Clinical Trial Data

### *Confirmation of Assessment Indices*

It is essential to determine the appropriate sample size when designing a clinical trial. The sample size is related to the specific assessment indices, which play a significant role in the trial results.

The confirmation methods for assessment indices include literature reviews, systematic reviews and meta‐analyses, with meta‐analyses being the most commonly applied. Meta‐analysis is a type of statistical method that integrates data from many studies with the same subject and specific conditions. This method of analysis can be applied to collect the index results for one specific disease from the literature, to analyze the rate of effectiveness and the rate of improvement using a particular type of medical device on the related disease, and, finally, to obtain the relative parameter range using historical data^7^.

The investigator should fully consider the detailed characteristics of the medical device product declared and a comparable product on the market. These characteristics are used to evaluate which database is the most appropriate for the assessment and comparison of data by meta‐analysis. The investigator should also state proper reasons for the selection. The most commonly used databases are scientific databases (e.g. China National Knowledge Internet, CNKI), clinical trial databases (e.g. Cochrane Central Register of Controlled Trials), system evaluation databases (e.g. Cochrane Library), and specialized databases (e.g. MEDION).

### *Determination of Non‐inferiority Margin*

Usually, the randomized non‐inferiority design is applied for the clinical trials of class III medical devices. The objective of these trials is to confirm that the difference between the curative effect (or safety) of the experimental group and control group medical device is no larger than the equivalent range that is previously set; that is, the difference is acceptable. The effectiveness and safety of the declared medical device should be established based on those of the control group device. The determination of the margin should be based on the situation in clinical practice^5^. Through meta‐analysis, the investigator can initially estimate the relative effect of the control group device, *M1*. After obtaining the appropriate rate of the control group effect, 1 *− f*, the non‐inferiority margin is determined as *M2* (*M2* = *f* × *M1*). The lower the *f* value, the more the effect of the experimental group device approaches that of the control group device^8^. Normally, the *f* value ranges from 0 to 0.5. The investigator should determine the prospective curative effect and the clinically recognized non‐inferiority margin of the control group device.

### *Estimation of the Sample Size*

The estimation of the sample size is a significant component of the clinical trial design, and is important for trial reliability. The investigator should adopt a classical statistical method and internationally recognized statistical software to estimate the sample size. The sample size adopted should meet the demands of the trial aim, and it is usually estimated using the major assessment index. The investigator should provide a statistical basis for determining the sample size. As for a randomized controlled trial, the statistical basis might include, but is not restricted to, the prospective curative effect (with the same conditions for the experimental and the control group), prospective differences between groups, significance level α, power β, the prospective rate of the lost follow‐up values, applied sample size equation, applied statistical software, and references.

According to *The Clinical Trial Designing Principle of Medical Devices*, as for the non‐inferiority trial, it is assumed that:

- The experimental and control group are randomly grouped at the ratio of 1:1
- The major assessment index is the prospective occurrence rate
- The variance is homogeneous and not approaching 0% or 100%.

The estimation formula of the sample size gives:

$$nT=n_{C}=\frac{{\left(Z_{1-\frac{\alpha }{2}}+Z_{1-\beta }\right)}^{2}\left[P_{C}\left(1-P_{C}\right)+P_{T}\left(1-P_{T}\right)\right]}{{\left(\left|D\right|-\Delta \right)}^{2}},$$

where n_*T*_ and n_*C*_ are, respectively, the sample sizes of the experimental group and the control group; $Z_{1-\frac{\alpha }{2}}$ and Z_1 − *β*_ are the fractional bits of the standardized normal distribution (when *α* = 0.05, $Z_{1-\frac{\alpha }{2}}=1.96$; when *β* = 0.2, Z_1 − *β*_ = 0.842, ${\left(Z_{1-\frac{\alpha }{2}}+Z_{1-\beta }\right)}^{2}=7.85$); P_*T*_ and P_*C*_ represent the prospective occurrence rates of the experimental group and the control group, respectively; |*D*| is the absolute difference between those two rates, that is, |*D*| = |P_*T*_ − P_*C*_|; and *Δ* is minus representing the non‐inferiority margin.

It is assumed that:

- The experimental and the control group are randomly grouped at the ratio of 1:1
- The major assessment index is a quantitative index
- The variance is homogeneous.

The estimation formula gives:

$$nT=n_{C}=\frac{2{\left(Z_{1-\frac{\alpha }{2}}+Z_{1-\beta }\right)}^{2}\sigma^{2}}{{\left(\left|D\right|-\Delta \right)}^{2}},$$

where *σ* is the prospective standard deviation of the control group; |*D*| is the absolute difference between two means, that is, |*D*| = |μ_*T*_ − μ_*C*_|; and *Δ* is a minus representing the non‐inferiority margin^9^.

## Clinical Trial Methods

### *Clinical Trial Methods*

Clinical trials can be categorized into various design methods, including parallel control, pairing, crossover and single‐group designs. The parallel control design is the most common among designs.

For a parallel control trial, a double‐blind study is usually applied, in which both the patient and the clinician do not recognize which group of products the patient is using. Sometimes, due to obvious differences in the appearances of medical devices being compared, the clinician might inevitably know the group division. In this case, a single‐blind study could be applied, and the clinician is obliged to keep the group division secret to patients. This method is applied to prevent the clinician and patients from focusing only on the performance of the experimental group, which might result in operative deviation and bias during the clinical trial^10^.

The randomized method is also applied for a parallel control trial, in which there is no specific division in grouping the patients. However, sometimes the superiority of the applied product has already been confirmed; then a non‐randomized trial can be adopted.

Normally, a multi‐center clinical trial is used to assess class III medical devices; that is, a few investigators conduct identical clinical trials using the same protocol in different centers. All centers begin and finish the trial simultaneously. The clinical trial report is drafted by the leading investigator. Many other studies also mention its viability^11^, ^12^.

According to *The Clinical Trial Designing Principle of Medical Devices*, in some cases a parallel control design is not available for an applied medical device. In that case, the other possible methods mentioned above could be further considered.

### *Inclusion Criteria and Exclusion Criteria*

Various class III medical devices might have different criteria for the patient to be included in or excluded from the clinical trial. The inclusion standards contain but are not restricted to patient age, gender, whether the body part at the site of the implanted medical device is well grown, whether the patient is available for the trial, and whether the patient or guardian would provide informed consent. The exclusion standards contain but are not restricted to the patient having allergies or a genetic disease, local infection of the body part at the site of the implanted medical device, use of drugs that are not proper for operation, intemperance, incompetence or incomprehensiveness of the study, and rejection of informed consent.

### *Follow‐up Period*

To obtain all the safety and effectiveness data for the declared medical devices, the follow‐up period of the clinical trial should either be longer than the degradation time of the product (if possible) or last until the reaction of the device with surrounding tissues becomes stable, for the CaP/CaSi bone‐filling materials. If the follow‐up period is 6 months, the follow‐up points should include at least 7 days, 3 months and 6 months.

The follow‐up content of the clinical trial is determined by the type of the medical device. It commonly contains information on wound healing and adverse reactions, subjective feelings of the patient, health examination, X‐ray film, and functional marking of the medical device. The content can be more specific for particular medical devices. For example, the follow‐up content for an artificial cochlea could include the free‐field hearing threshold, the MAIS questionnaire, and hepatorenal function examination; and that for a posterior spinal product for internal fixation can involve several scoring systems, such as JOA, ODI, SF‐36, and STS.

### *Data Collection and Analysis*

The data analysis should be based on various analysis sets, used to analyze different assessment indices, so that the test results can be completely and reasonably evaluated. The analysis sets usually contain a full analysis set, a per protocol set, and a safety set. The definitions of those analysis sets should be illustrated in the study report.

When analyzing the data, the investigator should consider the integrality of the data. All patients who have signed the informed consent and used the applied product should be included in the final statistical analyses. It should be illustrated in the trial protocol how the missing values of the major assessment index are filled and added when there is any lost case in the full analysis set. The sensitivity should be further analyzed to assess the effect of missing data on the stability of the result. The analyses of the major assessment index need to be conducted in both the full analyses set and the per protocol set. If those two sets provide a consistent conclusion, the reliability of the trial is promoted; otherwise, the investigator should fully discuss and explain any differences. The analysis of safety indices should be based on the safety set. The exclusion of data and the disposal of bias data should be carefully explained, and the criteria should be illustrated in the protocol in advance.

The information that must be clearly stated in the trial protocol includes the statistical type, the hypothesis, the threshold of clinical significance, the statistical analysis method, and the software used for the analysis. As for the major assessment index, the statistical results could be evaluated by point estimation, with a confidence interval of 95%. It is also important to perform the hypothesis test and calculate the relative *P* value on completing the statistical analyses, although the *P* value cannot be the only basis of the major index.

If two or more medical devices were implanted in one subject at multiple positions, the investigator needs to analyze the sensitivity of the results; that is, the cases and time of cases should be analyzed, respectively.

The investigator should list and describe all types of adverse reactions, as well as the degree of severity, frequencies and the connection to the experimental product during the clinical trial.

## Clinical Trial Study Announcements

### *Ethics and Informed Consent*

The investigator must comply with *The Declaration of Helsinki* and relative national clinical trial standards in conducting the clinical trial.

The clinical trial protocol should be approved by the medical ethics committee of the hospital where the study is conducted before the start of trial. The investigator is responsible for reporting to the ethics committee any safety issues for patients, adverse events and severe adverse events during the study. Any modification to the clinical protocol should be co‐authored by the sponsor and the investigator. The amendment should be submitted to the ethics committee. These procedures should be accomplished before the amendment goes into effect.

Before the patients are selected into the study, the clinician should discuss the aim, the procedure, and possible hazards with the patients or their designated representatives completely and comprehensively. The patients must give written informed consent, which should be saved for future reference^13^, ^14^.

### *Biases and Random Errors of the Clinical Trial*

The biases and random errors should be considered during the clinical trial design. The biases are obtained from systematic errors; hence, they should be reduced or avoided to the greatest extent possible. The random errors are affected by the sample size. Having an exceedingly large sample size might reduce random errors by providing more data. However, it may induce biases. This could make a clinically insignificant difference become statistically significant. Thus, when the investigator designs the clinical trial, it is essential to control the sample size, so that both the clinical and the statistical significance of the test results can be guaranteed^15^.

### *Conclusion*

In China, Class III medical devices are recognized as the medical devices with the highest risk to patients. Investigators must strictly assess the effectiveness and the safety of devices and scrupulously formulate the trial protocol. Assessment indices, clinical trial methods, and statistical methods should also be fully considered and selected. Furthermore, the clinical trial report should include clinical experiences, illustration, discussion and analysis of results, based on the trial designs and experimental data. These procedures are applied to obtain a detailed assessment of the effectiveness and the safety of the product, and to provide necessary conditions for the post‐market product application.
